# Pirfenidone reduces ovarian fibrosis and improves PCOS in letrozole-induced rat model

**DOI:** 10.17305/bb.2025.12676

**Published:** 2025-06-30

**Authors:** Ayşe Çakır Gündoğdu, Neziha Senem Arı, Ahmet Koçak, Gülnihal Şenol, Asiye Höbel, Ömer Eldiven, Fatih Kar, Orhan Özatik

**Affiliations:** 1 Department of Histology and Embryology, Faculty of Medicine, Kütahya Health Sciences University, Kütahya, Türkiye; 2 Department of Medical Biochemistry, Faculty of Medicine, Kütahya Health Sciences University, Kütahya, Türkiye

**Keywords:** Polycystic ovary syndrome, PCOS, ovarian fibrosis, pirfenidone, PFD, combined oral contraceptives, COCs, transforming growth factor-beta 1, TGF-β1

## Abstract

Polycystic ovary syndrome (PCOS) is a prevalent endocrine disorder characterized by cystic ovarian morphology, anovulation, and infertility. Ovarian fibrosis has recently emerged as a key pathological feature of PCOS. This study investigated whether pirfenidone (PFD), an antifibrotic agent, could improve ovarian dysfunction in a letrozole-induced PCOS rat model. Forty-two female Wistar albino rats were divided into six groups (*n* ═ 7 in each group): control, PFD, PCOS, PCOS/PFD, PCOS/combined oral contraceptives (COCs), and PCOS/PFD/COC. PCOS was induced using letrozole (1 mg/kg/day orally for 21 days). PFD (200 mg/kg/day) and/or COC (0.18 mg/kg cyproterone acetate and 0.00315 mg/kg ethinyl estradiol) were administered for 21 days. Compared to controls, PCOS rats exhibited significant disruptions in estrous cyclicity, ovarian morphology, and fibrosis-related markers (all *P <* 0.0001), despite no significant changes in testosterone (*P ═* 0.058) or estrogen (*P ═* 0.896) levels. PFD treatment significantly improved estrous cyclicity, follicular profile, and corpora lutea count (all *P <* 0.0001), reduced ovarian fibrosis (*P <* 0.0001), downregulated transforming growth factor-beta 1 (TGF-β1), connective tissue growth factor (CTGF), and matrix metallopeptidase (MMP)-9 (all *P <* 0.0001), and upregulated peroxisome proliferator-activated receptor-gamma (PPAR-γ) and MMP-2 (both *P <* 0.0001), without affecting hormone levels (*P ═* 0.945 and *P ═* 0.479, respectively). COC treatment also improved estrous cyclicity and ovarian histology (all *P <* 0.0001), reduced fibrosis (*P ═* 0.005), and modulated TGF-β1, CTGF, MMP-9, and PPAR-γ expression (*P ═* 0.0001 to <0.0001), but had no effect on MMP-2 (*P ═* 0.868). Combination therapy (PCOS/PFD/COC) provided additional improvement in corpora lutea count (*P <* 0.0001 vs PCOS/PFD) and collagen deposition (*P ═* 0.002 vs PCOS/PFD), but did not confer further benefits in fibrosis-related marker expression or folliculogenesis (all *P* > 0.05). These findings suggest that PFD mitigates PCOS pathology by targeting ovarian fibrosis, supporting antifibrotic therapy as a novel and promising approach.

## Introduction

Polycystic ovary syndrome (PCOS) is a prevalent endocrine and metabolic disorder affecting 11%–13% of women of reproductive age. It is characterized by hyperandrogenism, oligo- or anovulation, menstrual irregularities, and the presence of multiple ovarian cysts [1]. Beyond hormonal and metabolic disturbances, PCOS also exhibits fibrotic features marked by structural changes in the ovarian stroma. Ovarian fibrosis significantly contributes to impaired folliculogenesis and reproductive dysfunction [2]. In PCOS, hyperandrogenism and insulin resistance drive chronic low-grade inflammation and heightened oxidative stress, both of which disrupt normal follicular maturation [3]. These pathological conditions promote the overexpression of pro-inflammatory cytokines such as tumor necrosis factor-alpha (TNF-α) and interleukin-6 (IL-6) in granulosa cells, leading to fibroblast activation and excessive collagen deposition in the ovarian stroma [3, 4]. This fibrotic remodeling is closely associated with elevated levels of pro-fibrotic mediators like transforming growth factor-beta 1 (TGF-β1) and connective tissue growth factor (CTGF), which are upregulated under hyperandrogenic conditions [5]. Additionally, oxidative stress and chronic inflammation further exacerbate the fibrotic cascade by promoting fibroblast activation and altering the expression of matrix metalloproteinases (MMPs) and their inhibitors (TIMPs), thereby disturbing extracellular matrix (ECM) turnover [6]. Simultaneously, reduced expression or activity of the anti-fibrotic nuclear receptor peroxisome proliferator-activated receptor-gamma (PPAR-γ) removes a key regulatory brake on TGF-β signaling and matrix remodeling, amplifying collagen accumulation in the stroma [7]. Thus, ovarian fibrosis is not merely a secondary consequence but a central, often underrecognized component of the PCOS phenotype—disrupting ovarian architecture and impairing ovulation and oocyte quality. This underscores the need for therapeutic strategies that specifically target stromal fibrosis, a factor currently overlooked in standard PCOS management. Combined oral contraceptives (COCs) are the first-line pharmacological treatment for PCOS, particularly for patients with menstrual irregularities, clinical hyperandrogenism, and acne. COCs suppress ovulation and luteinizing hormone (LH) secretion while increasing sex hormone-binding globulin (SHBG), thereby reducing free androgen levels and alleviating symptoms [8]. However, despite their endocrine benefits, growing evidence suggests that COCs do not address the broader metabolic and structural abnormalities associated with PCOS [9]. In particular, there is no evidence that COCs influence ovarian fibrotic remodeling—an increasingly recognized pathological feature. This limitation has prompted the search for adjunctive or alternative treatments that can complement hormonal regulation by targeting the fibrotic aspects of PCOS. Pirfenidone (PFD) is an antifibrotic and anti-inflammatory agent currently approved for treating idiopathic pulmonary fibrosis (IPF). It targets TGF-β–mediated signaling pathways, inhibiting fibroblast proliferation, collagen synthesis, and myofibroblast differentiation, while also reducing oxidative stress and pro-inflammatory cytokine activity—thereby interrupting key fibrotic mechanisms [10]. Preclinical studies have demonstrated PFD’s efficacy in various fibrotic conditions, including those affecting the liver, kidneys, heart, pancreas, and bladder [11–14]. The first evidence of PFD’s antifibrotic effect on ovarian tissue came from Umehara et al., [15] who demonstrated that stromal fibrosis disrupts oocyte release, and that PFD treatment reverses collagen accumulation and restores ovulatory capacity in reproductively aged and obese mice. More recently, Amargant et al. (2025) [16] reported that PFD attenuated age-related ovarian fibrosis, increased follicle and corpora lutea counts, and improved estrous cyclicity in aged mice. These findings suggest that modulating the fibroinflammatory ovarian microenvironment with PFD may enhance ovarian function and reproductive outcomes. However, PFD’s potential in treating PCOS-related ovarian fibrosis remains unexplored—a critical gap in fibrotic disease research, particularly as fibrosis gains recognition as a central pathological feature of PCOS. In this study, we aimed to evaluate the therapeutic effects of PFD, alone or in combination with COCs, on ovarian fibrosis in a letrozole-induced rat model of PCOS. We assessed histological architecture, hormone levels, and the immunohistochemical expression of fibrosis-related markers—including TGF-β1, CTGF, PPAR-γ, MMP-2, and MMP-9—to determine whether PFD can ameliorate fibrotic changes and improve ovarian integrity and hormonal balance. This work addresses a critical gap in PCOS research and proposes a novel antifibrotic strategy that may complement current hormone-based therapies.

## Materials and methods

### Ethical considerations and experimental procedures on animals

**Figure 1.**
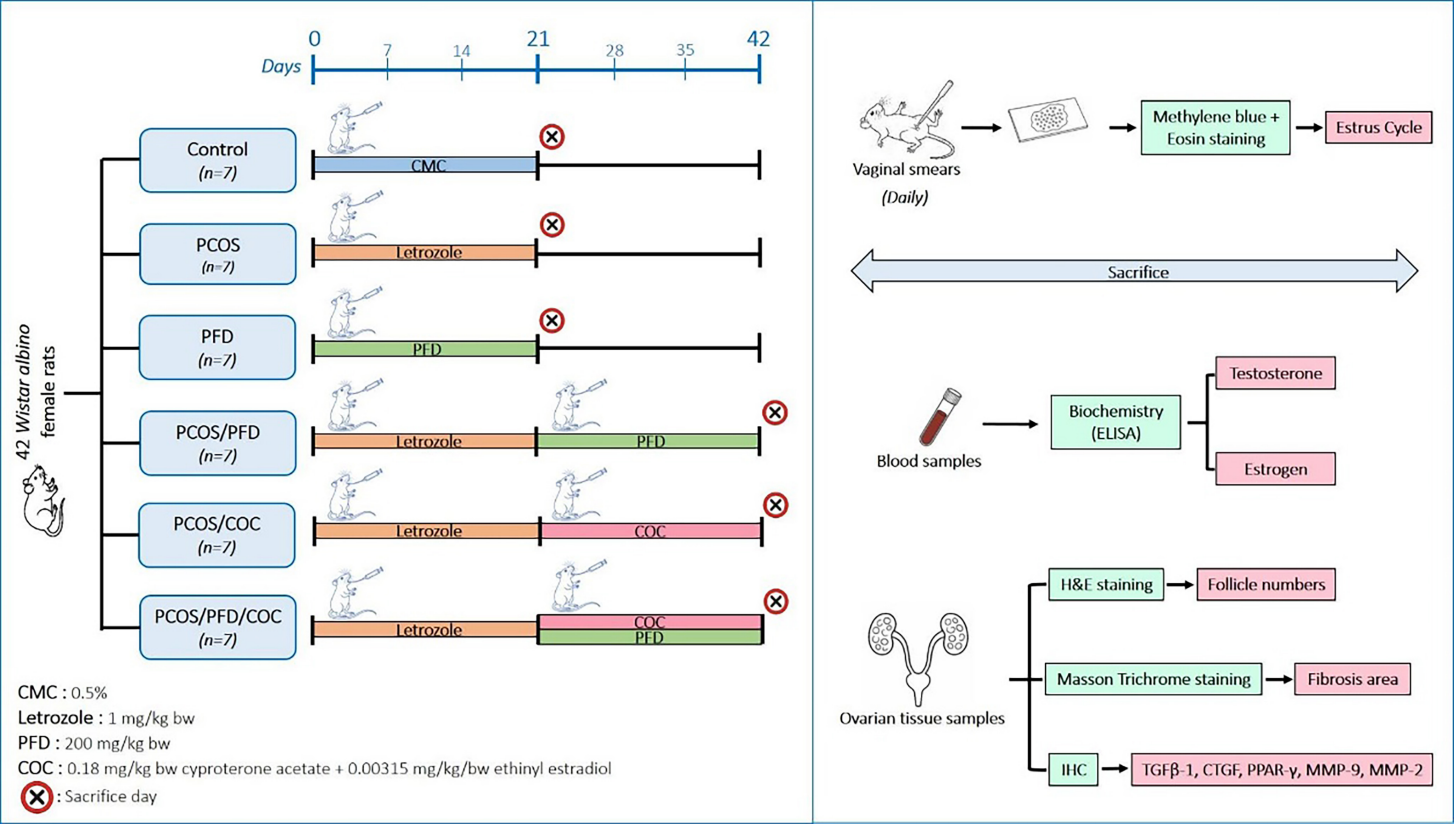
**Flowchart of the experimental design. Schematic representation of group allocation, treatment protocols, duration of the experiment, and sampling time points in the study.** Forty-two female *Wistar albino* rats were randomly divided into six groups (Control, PFD, PCOS, PCOS/PFD, PCOS/COC, PCOS/PFD/COC). Treatments were administered daily for 21 days following PCOS induction with letrozole. Vaginal cytology, histological, biochemical, and immunohistochemical analyses were performed as outlined. PCOS: Polycystic ovarian syndrome; PFD: Pirfenidone; COC: Combined oral contraceptives.

Forty-two female Wistar albino rats (3–4 months old, 250–300 g) were obtained from the Experimental Animals Breeding and Research Unit (DEHYUB) at Kütahya Health Sciences University. All experimental procedures were approved by the Institutional Animal Ethics Committee (HADYEK; protocol number 2023.06.04). The animals were housed under standard laboratory conditions (12 h light/12 h dark cycle, 22 ± 2 ^∘^C) with ad libitum access to food and water. Rats were randomly assigned to six groups (*n* ═ 7 per group): control, PFD, PCOS, PCOS + PFD (PCOS/PFD), PCOS + COC (PCOS/COC), and PCOS + PFD + COC (PCOS/PFD/COC). To induce PCOS, the last four groups received 1 mg/kg letrozole (Femara, Novartis), dissolved in 0.5% carboxymethyl cellulose (CMC; Carl Roth, 9004-32-4), via oral gavage once daily for 21 days, as previously described [17]. The control group received only CMC, while the PFD group received 200 mg/kg PFD (Pirfect, Nobel) during the same period [18]. Following successful induction, the final three groups received further treatment for 21 days with 200 mg/kg PFD and/or Diane-35 (Bayer), which contains 0.18 mg/kg cyproterone acetate and 0.00315 mg/kg ethinyl estradiol [19]. All drug solutions were freshly prepared and administered daily via oral gavage. A visual summary of the study design is presented in Figure 1. Body weights were recorded weekly throughout the study. At the end of the treatment period, animals were weighed and deeply anesthetized with ketamine (90 mg/kg) and xylazine (10 mg/kg) prior to intracardiac blood collection. Bilateral ovaries were then excised and weighed. In this study, PFD was administered orally at 200 mg/kg/day for 21 days. This dosage was based on preclinical evidence supporting its antifibrotic efficacy and tolerability in rodents. In pulmonary fibrosis models, such as those induced by bleomycin or PM10, PFD at this dose significantly reduced collagen deposition, TGF-β1 activation, and inflammatory markers without observable systemic toxicity [20, 21]. Regulatory toxicology assessments have also shown that rats receiving up to 200 mg/kg/day exhibit only mild hepatocellular hypertrophy and enzyme elevations, with no clinical signs of toxicity, supporting this dose as the no observed adverse effect level (NOAEL) in rodents [22].

### Analysis of the estrous cycle

Vaginal smears were collected daily between 10:00 and 13:00 throughout the study. Approximately 200 µL of sterile saline was gently instilled into the vaginal cavity using a pipette, followed by 2–3 gentle flushes to dislodge epithelial cells. The resulting suspension was transferred onto microscope slides and air-dried. For cytological evaluation, slides were sequentially stained with 0.1% methylene blue for 3 min, rinsed in distilled water for 1 minute, and counterstained with 1% eosin for 2 min. After a final rinse, the slides were mounted with glycerol and examined under a Nikon Eclipse 80i light microscope (Nikon, Germany) at 200× magnification. The stages of the estrous cycle—proestrus, estrus, metestrus, and diestrus—were identified based on standard cytological criteria.

### Biochemical analysis

Blood samples were collected in serum-separator tubes (SSTs) and allowed to clot at room temperature. The samples were then centrifuged at 4000 rpm for 5 min (MSE Mistral 2000, France). The resulting serum was aliquoted into polyethylene tubes and stored at −80 ^∘^C until further analysis. Serum testosterone and estrogen concentrations were quantified using commercially available sandwich ELISA kits (Testosterone: E00259Ra; Estrogen: E0176Ra; BT Laboratory, Korea) according to the manufacturer’s instructions. Optical density was measured using a Beckman Coulter AU680 analyzer (Beckman Coulter, Miami, FL, USA).

### Hematoxylin and eosin (H&E) staining

Ovaries, sectioned along their longest longitudinal axis, were fixed in 10% (v/v) formaldehyde for 48 h. Following standard tissue processing, paraffin-embedded blocks were prepared, and serial sections of 4 µm thickness were obtained using a microtome. The sections were deparaffinized in xylene, rehydrated through a descending ethanol series (100%–70%), and finally rinsed in water. Hematoxylin staining was performed for 10 min, followed by eosin staining for 3 min. The sections were then dehydrated through an ascending ethanol series (70%–100%), cleared in xylene, and mounted with Entellan. Using a Zeiss Calibri 7 light microscope, preantral and antral follicles, corpora lutea, and cystic follicles were counted in five sections (taken at intervals of every fifth section) from both ovaries of each animal.

### Masson trichrome staining

To assess the extent of fibrosis, tissue sections were stained with Masson’s trichrome using a commercial kit (5022, GBL, İstanbul, Türkiye). Briefly, following standard tissue processing, 4 µm-thick sections were prepared and deparaffinized. The sections were then sequentially stained with Weigert’s iron hematoxylin, picric acid, Biebrich scarlet-acid fuchsin, phosphomolybdic acid, and aniline blue. After dehydration and clearing, the slides were mounted with Entellan and imaged using a Zeiss Calibri 7 light microscope. Fibrosis quantification was performed using ImageJ/Fiji v1.52 software (NIH, USA) by calculating the percentage of blue-stained (fibrotic) area relative to the total tissue area in each image. A minimum of five non-overlapping fields per section were analyzed.

**Figure 2.**
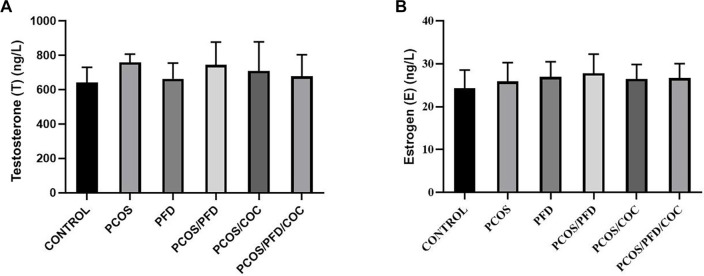
**Letrozole and PFD do not significantly alter serum testosterone (T) and estrogen (E) levels.** Serum levels of T (A) and E (B) were measured using ELISA kits, as described in the materials and methods. Neither the induction of PCOS with letrozole nor treatment with PFD and/or COC significantly altered serum T and E levels (*P* > 0.05). Data are presented as mean ± SD (*n* ═ 7 per group). PCOS: Polycystic ovarian syndrome; PFD: Pirfenidone; COC: Combined oral contraceptives; SD: Standard deviation.

### Immunohistochemistry

Ovarian tissue sections (4 µm thick) were first incubated with 3% hydrogen peroxide (H_2_O_2_) for 15 min to block endogenous peroxidase activity. Antigen retrieval was performed via three cycles of microwave heating in citrate buffer (1X; pH 6.0; Thermo Fisher Scientific, TA-9003-999). After cooling, sections were treated with Ultra V Block (Thermo Fisher Scientific, TP-125-HL) for 10 min to prevent non-specific binding. Samples were then incubated overnight at 4 ^∘^C with primary antibodies targeting TGFβ-1 (1:200; Santa Cruz Biotechnology, sc-130348), CTGF (1:100; BT Lab, BT-AP08174), PPAR-γ (1:200; ABclonal, A11183), MMP-9 (1:100; ABclonal, A0289), or MMP-2 (1:200; Santa Cruz Biotechnology, sc-13595). Following PBS washes, sections were incubated with an HRP-conjugated goat anti-rabbit secondary antibody (Thermo Fisher Scientific, TP-125-HL) at room temperature for one hour. Immunoreactivity was visualized using the 3,3-diaminobenzidine (DAB) chromogen (Thermo Fisher Scientific, TA-125-HD). Slides were mounted with Entellan and examined under a Zeiss Calibri 7 light microscope. For semiquantitative analysis, five randomly selected non-overlapping fields per section were analyzed at 400× magnification. A minimum of 100 cells per field were evaluated in the theca and stromal compartments, as fibrotic activity was predominantly localized in these regions. Granulosa cells were excluded from scoring. The histological score (H-score) was calculated using the formula ΣPi(i + 1), where i denotes staining intensity (0 ═ none, 1 ═ weak, 2 ═ moderate, 3 ═ strong), and Pi represents the percentage of positively stained cells. This method ensured consistent assessment of fibrosis-related staining across the relevant ovarian compartments.

### Statistical analysis

An a priori power analysis was conducted using G*Power software (version 3.1.9.7, Düsseldorf, Germany) to determine the minimum sample size needed to detect statistically significant differences among six groups. Based on a medium effect size (*f* ═ 0.4), a significance level (α) of 0.05, and a power (1–β) of 0.95, the required total sample size was calculated to be 45. To maintain equal group sizes, the final sample size was set at 42 (*n* ═ 7 per group), yielding a power of 0.93. Study data are presented as mean ± standard deviation (SD). Statistical analyses were performed using SPSS version 20.0 for Windows (IBM Corp., Armonk, NY, USA). Normality was assessed prior to analysis. Parametric data were analyzed using one-way analysis of variance (ANOVA) followed by Tukey’s post hoc test, while non-parametric data were evaluated using the Kruskal–Wallis one-way ANOVA. Statistical significance was determined based on *P* values, with differences considered significant at *P* < 0.05.

## Results

### Serum testosterone and estrogen levels remain unaffected by letrozole and subsequent treatments

To evaluate whether PCOS induction and subsequent treatments affected systemic sex hormone levels, serum testosterone and estrogen concentrations were measured (Figure 2). Testosterone levels exhibited a trend toward elevation in the PCOS group (759.41 ± 47.62 ng/L) compared to the control group (640.90 ± 89.23 ng/L), although this difference did not reach statistical significance (*P* ═ 0.058). In healthy rats, treatment with PFD alone yielded testosterone levels (662.77 ± 92.39 ng/L) comparable to the control group (*P* ═ 0.968). Among PCOS rats, administration of either PFD (744.46 ± 132.57 ng/L) or COC (709.62 ± 169.24 ng/L) did not significantly reduce testosterone levels relative to the untreated PCOS group (*P* ═ 0.945 and *P* ═ 0.765, respectively). The combination of PFD and COC in PCOS rats resulted in a mean testosterone level of 677.20 ± 126.60 ng/L, which also did not significantly differ from levels observed in the PCOS/PFD or PCOS/COC groups (*P* ═ 0.750 and *P* ═ 0.979, respectively). Similarly, serum estrogen concentrations did not significantly differ among experimental groups. The control group had a mean estrogen level of 24.26 ± 4.30 ng/L, while the PCOS group showed a comparable level of 25.83 ± 4.44 ng/L (*P* ═ 0.896). PFD treatment alone resulted in a mean level of 26.97 ± 3.52 ng/L (*P* ═ 0.459 vs control). Estrogen levels in the PCOS/PFD and PCOS/COC groups were 27.81 ± 4.45 ng/L (*P* ═ 0.479 vs PCOS) and 26.45 ± 3.39 ng/L (*P* ═ 0.681 vs PCOS), respectively. The PCOS/PFD/COC group exhibited a mean estrogen concentration of 26.69 ± 4.42 ng/L (*P* ═ 0.903 vs PCOS). Collectively, these findings suggest that none of the treatments significantly altered systemic estrogen levels.

### PFD ameliorates disruptions in the estrous cycle

**Figure 3.**
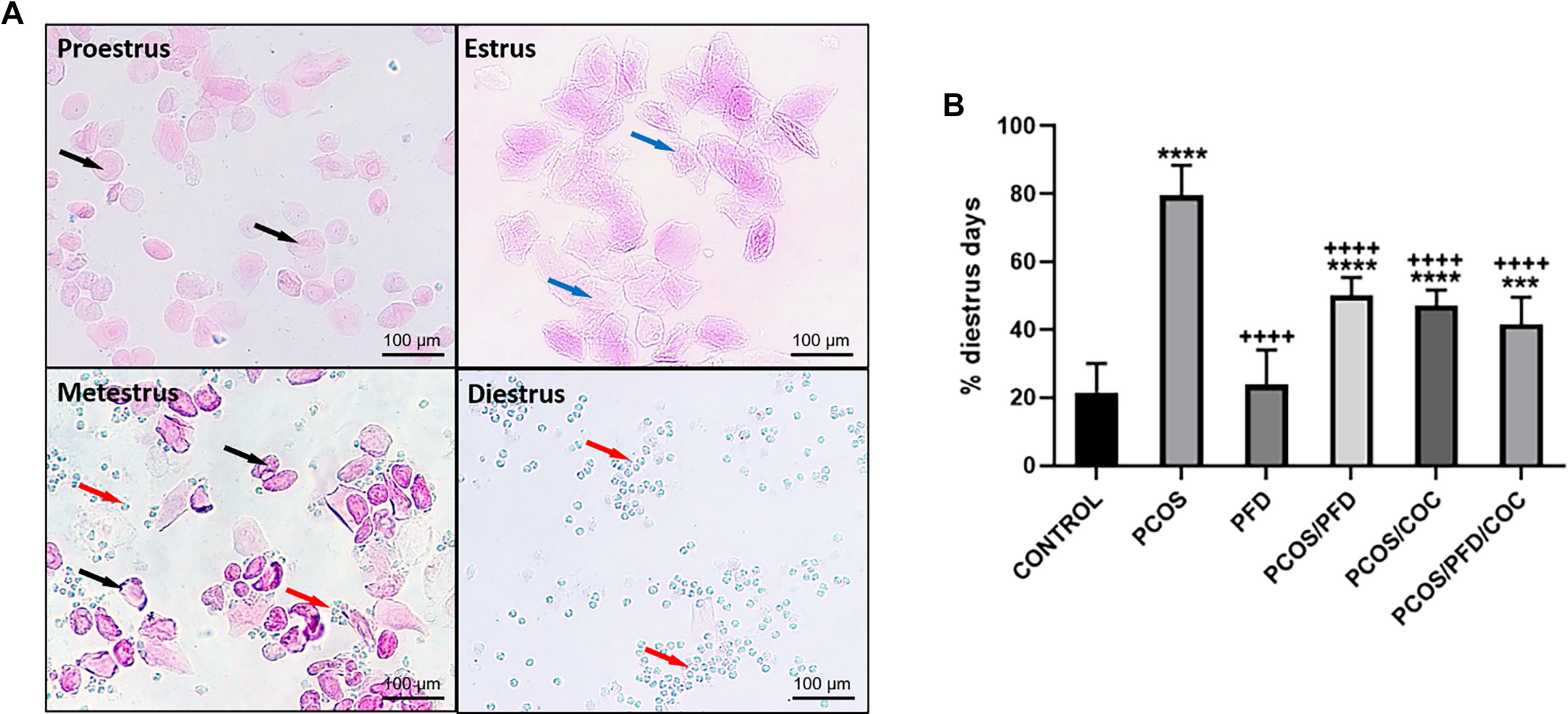
**PFD enables rats with PCOS to exit the diestrus phase.** (A) Methylene blue and eosin stained vaginal smears showing the different phases of the estrous cycle. Nucleated epithelial cells (black arrows), cornified epithelial cells (blue arrows), and leukocytes (red arrows). Scale bars ═ 100 µm. (B) Comparison of the percentage of diestrus days between groups. Data are expressed as mean ± SD (*n* ═ 7 per group). ^***^P < 0.001, ^****^P < 0.0001 vs Control; ^++++^*P <* 0.0001 vs PCOS. PCOS: Polycystic ovarian syndrome; PFD: Pirfenidone; COC: Combined oral contraceptives; SD: Standard deviation.

Daily vaginal smears were evaluated using methylene blue and eosin staining to determine estrous cycle stages, based on the relative proportions of nucleated epithelial cells, cornified epithelial cells, and leukocytes. As shown in Figure 3A, proestrus was characterized by a predominance of nucleated epithelial cells; estrus by cornified epithelial cells; metestrus by a mix of cornified epithelial cells and leukocytes; and diestrus by a predominance of leukocytes. Letrozole treatment in the PCOS group induced a persistent diestrus phase following the initial estrus, with no transition to proestrus observed throughout the experimental period. The percentage of days spent in diestrus was significantly higher in the PCOS group (79.58 ± 8.75%) compared to the control group (21.52 ± 8.62%, *P* < 0.0001). Treatment with PFD in PCOS rats (PCOS/PFD) significantly reduced the duration of diestrus to 50.12 ± 5.18% (*P* < 0.0001 vs PCOS). Similarly, the PCOS/COC (47.07 ± 4.67%) and PCOS/PFD/COC (41.57 ± 7.99%) groups showed substantial improvements compared to the PCOS group (*P* < 0.0001 for both). No significant difference was observed between the combination therapy group (PCOS/PFD/COC) and the monotherapy groups (PCOS/PFD: *P* ═ 0.965; PCOS/COC: *P* ═ 0.989), suggesting that the addition of PFD to COC did not provide further benefit in restoring the estrous cycle. In healthy rats, PFD treatment (PFD group, 23.62 ± 10.41%) resulted in diestrus durations comparable to controls (*P* ═ 0.999), indicating no adverse effects on the normal estrous cycle. Groupwise comparisons are illustrated in Figure 3B.

**Figure 4.**
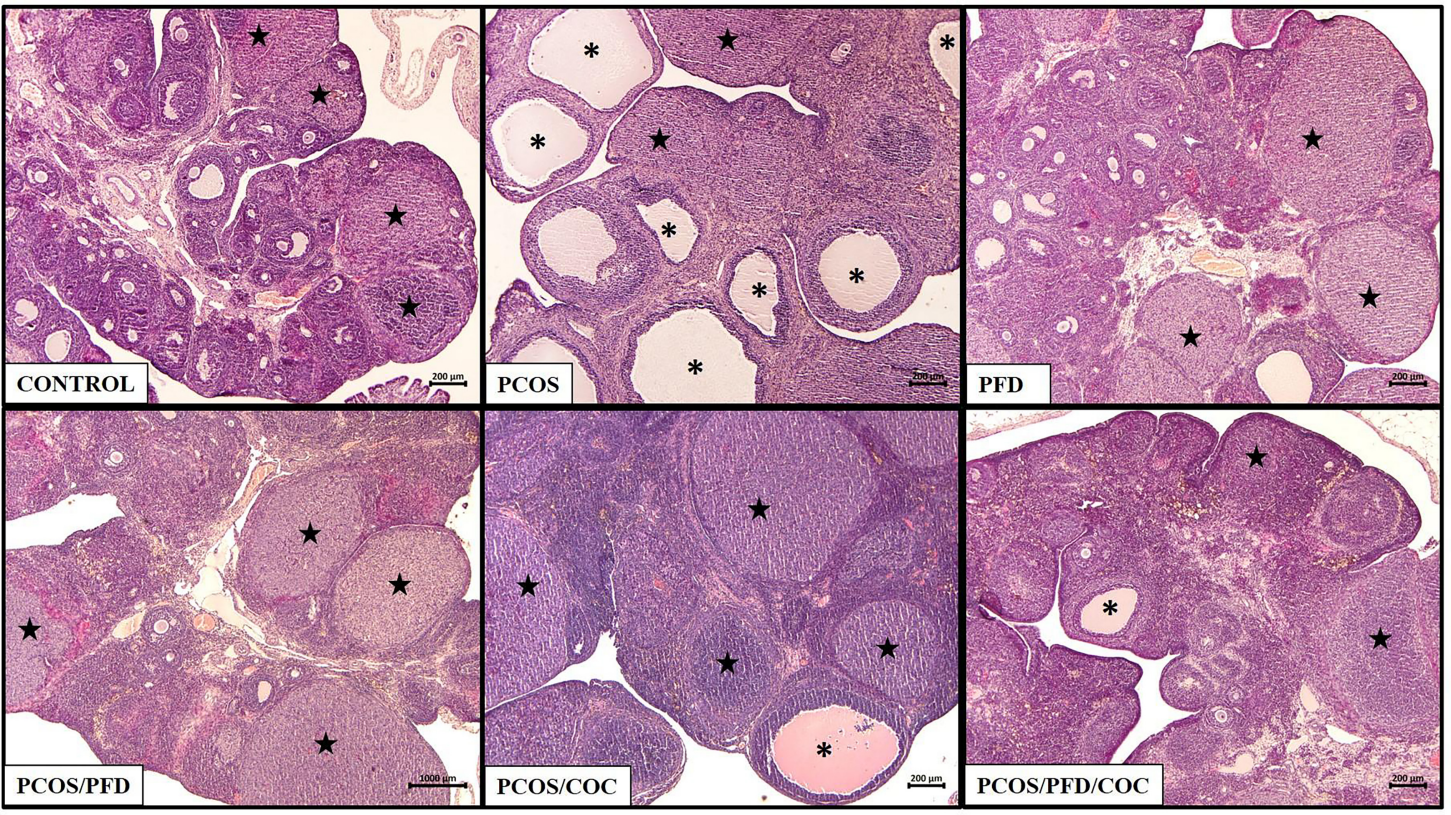
**PFD ameliorates ovarian histopathology in rats with PCOS.** Histopathological assessment of H&E-stained ovarian tissues. The Control group displays normal ovarian morphology. Numerous cystic follicles (CFs; asterisks) are observed in the letrozole-induced PCOS group. The PFD-treated group shows preserved normal histology. In the PCOS/PFD group, CFs are reduced while corpora lutea (CLs; stars) are increased compared to the PCOS group. The PCOS/COC group exhibits a reduction in CFs without a notable change in CLs. In the PCOS/PFD/COC group, both a decrease in CFs and an increase in CLs are evident. Scale bars ═ 200 µm. PCOS: Polycystic ovarian syndrome; PFD: Pirfenidone; COC: Combined oral contraceptives; H&E: Hematoxylin and eosin.

**Table 1:** Ovarian morphological parameters in control, PCOS, and treatment groups

**Variables**	**Control**	**PCOS**	**PFD**	**PCOS/PFD**	**PCOS/COC**	**PCOS/PFD/COC**
Ovarian weight (mg/100g)	39.18 ± 7.37	36.79 ± 3.96	39.26 ± 5.63	38.61 ± 7.26	35.00 ± 5.99	39.74 ± 7.95
Preantral follicle (*n*)	9.60 ± 1.35	18.89 ± 2.56^a^	10.74 ± 1.29^b^	14.26 ± 2.11^ab^	16.31 ± 1.89^bc^	14.83 ± 2.38^abd^
Antral follicle (*n*)	6.83 ± 1.29	14.89 ± 2.35^a^	7.14 ± 1.17^b^	9.91 ± 1.76^ab^	13.00 ± 2.00^bc^	12.49 ± 1.70^ab^
Cystic follicle (*n*)	1.40 ± 0.85	23.40 ± 3.96^a^	1.71 ± 0.96^b^	2.80 ± 1.05^b^	5.11 ± 2.25^bc^	3.46 ± 1.80^abd^
Total follicle (*n*)	17.31 ± 2.11	58.23 ± 4.15^a^	19.57 ± 2.23^ab^	27.34 ± 2.67^ab^	34.77 ± 3.11^bc^	30.91 ± 3.38^abd^
Corpara lutea (*n*)	7.71 ± 1.23	1.83 ± 1.72^a^	7.86 ± 0.88^b^	7.63 ± 1.44^b^	3.60 ± 1.87^bc^	4.71 ± 1.43^abd^

Data are shown as mean ± SD. Superscript letters indicate significant differences vs specified groups (ANOVA with Tukey post hoc, *P* < 0.05): a: Control, b: PCOS, c: PCOS/PFD, d: PCOS/COC. PCOS: Polycystic ovary syndrome; PFD: Pirfenidone; COC: Combined oral contraceptive; SD: Standard deviation; ANOVA: Analysis of variance.

**Figure 5.**
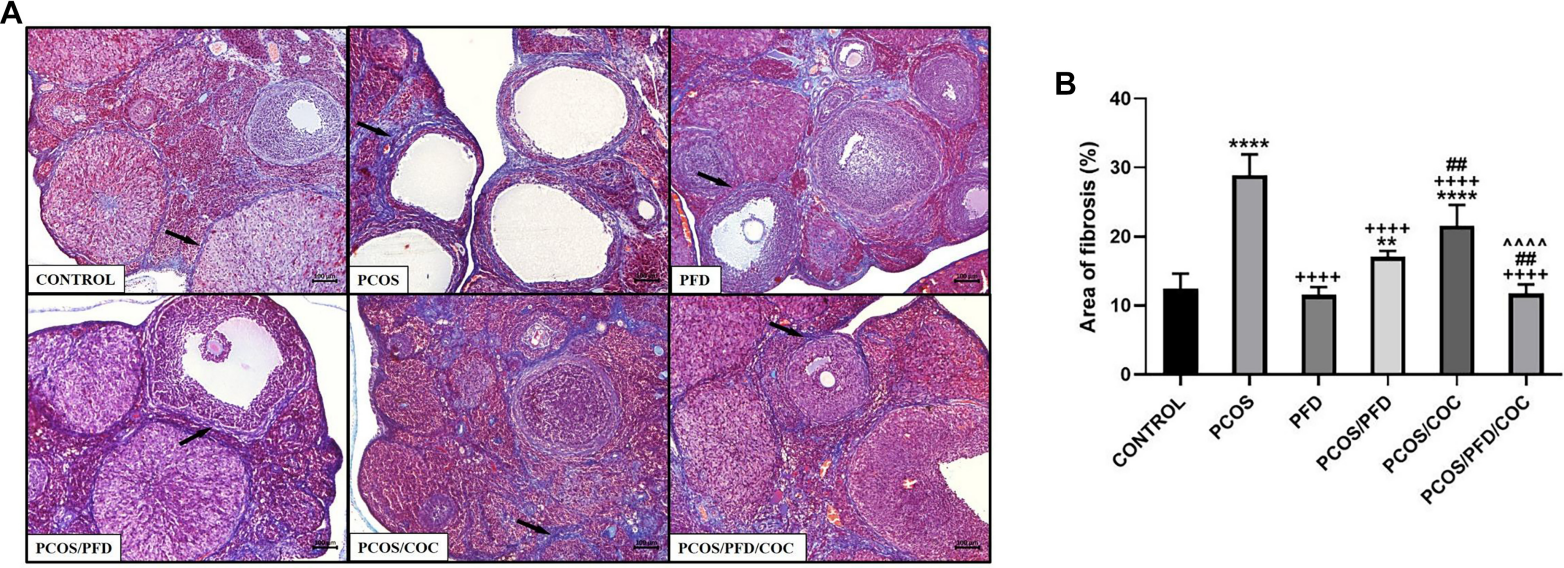
**PFD ameliorates ovarian fibrosis in rats with PCOS.** (A) Histopathological examination of ovarian fibrosis in tissues stained with Masson trichrome. Arrows indicate collagen deposits around the follicles. Scale bars ═ 200 µm. (B) Comparison of the percentage of fibrosis area among groups (mean ± SD; *n* ═ 7). **P < 0.01 ****P < 0.0001 vs control; ^++++^*P <* 0.0001 vs PCOS; ^##^*P <* 0.01 vs PCOS/PFD; ^ˆˆˆˆ^*P <* 0.0001 vs PCOS/COC. PCOS: Polycystic ovarian syndrome; PFD: Pirfenidone; COC: Combined oral contraceptives; SD: Standard deviation.

### PFD improves ovarian morphology in rats with PCOS

Histological analysis of ovarian sections (Figure 4) revealed distinct morphological patterns across the experimental groups, highlighting the effects of PCOS induction and subsequent treatments. In the control group, ovarian sections displayed multiple follicles at various developmental stages and numerous corpora lutea, consistent with normal ovulatory activity. Antral follicles exhibited 8–10 layers of granulosa cells, a well-formed antrum, and normal theca thickness. In contrast, the PCOS group showed classic polycystic morphology, characterized by numerous large cystic follicles lacking oocytes, a reduced granulosa layer (2–3 layers), and intact theca layers. The PFD-treated group maintained a histoarchitecture similar to controls, with healthy follicular structures and visible corpora lutea. In the PCOS/PFD group, fewer cystic follicles and increased corpora lutea indicated partial restoration of ovulatory activity. Likewise, the PCOS/COC group demonstrated reduced cystic structures and partially organized follicular development, although the relatively low number of corpora lutea may suggest a limited or delayed ovulatory response. The PCOS/PFD/COC group exhibited the most normalized histological profile, with a balanced distribution of follicular types, fewer cystic follicles, and enhanced luteinization, indicating improved ovarian recovery. These observations were quantitatively supported by morphometric analysis (Table 1). The PCOS group exhibited significant morphological alterations compared to controls. Ovarian weight was slightly lower (36.79 ± 3.96 mg/100 g vs 39.18 ± 7.37 mg/100 g), though the difference was not statistically significant. However, all follicular parameters were markedly affected. The PCOS group showed significantly higher numbers of preantral (18.89 ± 2.56 vs 9.60 ± 1.35, *P* < 0.0001), antral (14.89 ± 2.33 vs 6.83 ± 1.29, *P* < 0.0001), cystic (23.40 ± 3.96 vs 1.40 ± 0.85, *P* < 0.0001), and total follicles (58.23 ± 4.15 vs 17.31 ± 2.11, *P* < 0.0001), alongside a significant reduction in corpora lutea (1.83 ± 1.72 vs 7.71 ± 1.23, *P* < 0.0001), indicating anovulation. PFD treatment alone did not produce significant differences from the control group in any parameter, suggesting it has a neutral effect on normal ovarian morphology. The PCOS/PFD group, however, showed significant reductions in preantral (14.26 ± 2.11, *P* < 0.0001), antral (9.91 ± 1.76, *P* < 0.0001), cystic (2.80 ± 1.05, *P* < 0.0001), and total follicles (27.34 ± 2.67, *P* < 0.0001) compared to the PCOS group, along with a marked increase in corpora lutea (7.63 ± 1.44, *P* < 0.0001), reflecting partial restoration of ovulatory function. In the PCOS/COC group, treatment led to improvements in preantral (16.31 ± 1.89, *P* < 0.0001), antral (13.00 ± 2.00, *P* ═ 0.007), cystic (5.11 ± 2.20, *P* < 0.0001), and total follicle counts (34.77 ± 3.11, *P* < 0.0001) compared to the PCOS group. However, corpora lutea remained relatively low (3.60 ± 1.87, *P* ═ 0.005), suggesting a less pronounced ovulatory response. Comparative analysis between the PCOS/PFD and PCOS/COC groups revealed significant differences in several parameters. The PCOS/PFD group had significantly fewer cystic follicles (2.80 ± 1.05 vs 5.11 ± 2.25, *P* ═ 0.0001) and a lower total follicle count (27.34 ± 2.67 vs 34.77 ± 3.11, *P* ═ 0.005), indicating a more normalized follicular reserve. It also had significantly more corpora lutea (7.63 ± 1.44 vs 3.60 ± 1.87, *P* < 0.0001), suggesting greater ovulatory restoration. Differences in preantral (14.26 ± 2.11 vs 16.31 ± 1.89, *P* ═ 0.058) and antral follicles (9.91 ± 1.76 vs 13.00 ± 2.00, *P* ═ 0.058) were not statistically significant but trended higher in the PCOS/COC group. The combination therapy group (PCOS/PFD/COC) exhibited the most favorable morphological outcomes. Compared to the PCOS group, it showed significant improvement in all follicular parameters (*P* < 0.0001 for all). Relative to the PCOS/PFD group, the combination group had higher numbers of corpora lutea (7.63 ± 1.44 vs 4.71 ± 1.43, *P* < 0.0001) and a more normalized total follicle count (30.91 ± 3.38 vs 27.34 ± 2.67, *P* < 0.0001), though cystic follicle numbers did not differ significantly (3.46 ± 1.80 vs 2.80 ± 1.05, *P* ═ 0.78). Compared to the PCOS/COC group, the combination therapy produced superior results, with more corpora lutea (4.71 ± 1.43 vs 3.60 ± 1.87, *P* ═ 0.020), fewer cystic follicles (3.46 ± 1.80 vs 5.11 ± 2.25, *P* ═ 0.015), and a reduced total follicle count (30.91 ± 3.38 vs 34.77 ± 3.11, *P* < 0.0001), indicating a more robust restoration of ovarian function.

**Figure 6.**
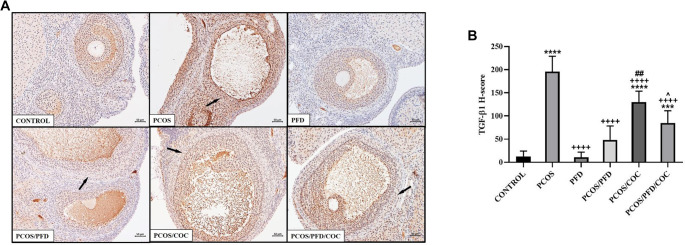
**PFD attenuates TGF-β1 expression in the ovarian stroma of PCOS rats.** (A) Representative immunohistochemical images of TGF-β1 expression in ovarian sections. Arrows indicate TGF-β1-positive areas in the stromal tissue surrounding the follicles. Scale bars ═ 200 µm. (B) Comparison of TGF-β1 H-scores among groups (mean ± SD; *n* ═ 7). ***P < 0.001, ****P < 0.0001 vs Control; ^++++^*P <* 0.0001 vs PCOS; ^##^*P <* 0.01 vs PCOS/PFD; ^ˆ^*P <* 0.05 vs PCOS/COC. PCOS: Polycystic ovarian syndrome; PFD: Pirfenidone; COC: Combined oral contraceptives; TGF-β1: Transforming growth factor-beta 1; SD: Standard deviation.

**Figure 7.**
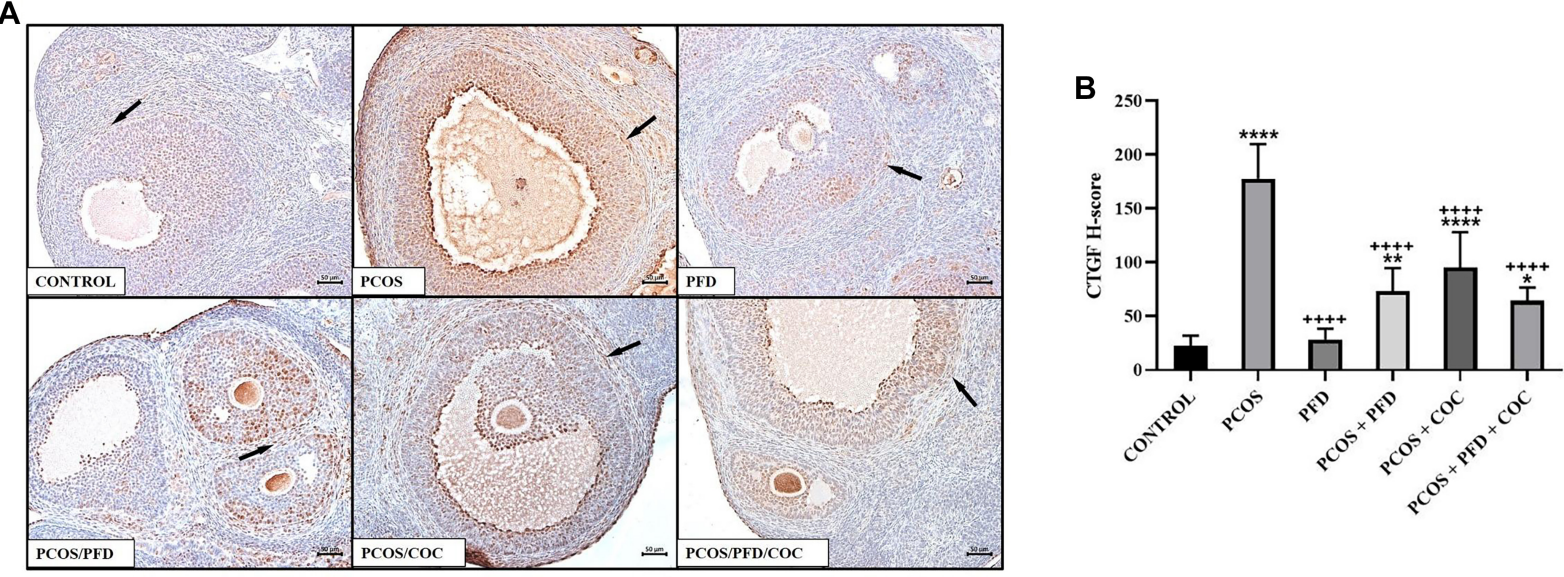
**PFD attenuates CTGF expression in the ovarian stroma of rats with PCOS.** (A) Representative immunohistochemical images of CTGF expression in ovarian sections. Arrows indicate CTGF-positive areas in the stromal tissue surrounding the follicles. Scale bars ═ 200µm. (B) Comparison of CTGF H-scores among groups (mean ± SD; *n* ═ 7). ^*^P < 0.05, **P < 0.01, ****P < 0.0001 vs Control; ^++++^*P <* 0.0001 vs PCOS. PCOS: Polycystic ovarian syndrome; PFD: Pirfenidone; COC: Combined oral contraceptives; CTGF: Connective tissue growth factor; SD: Standard deviation.

**Figure 8.**
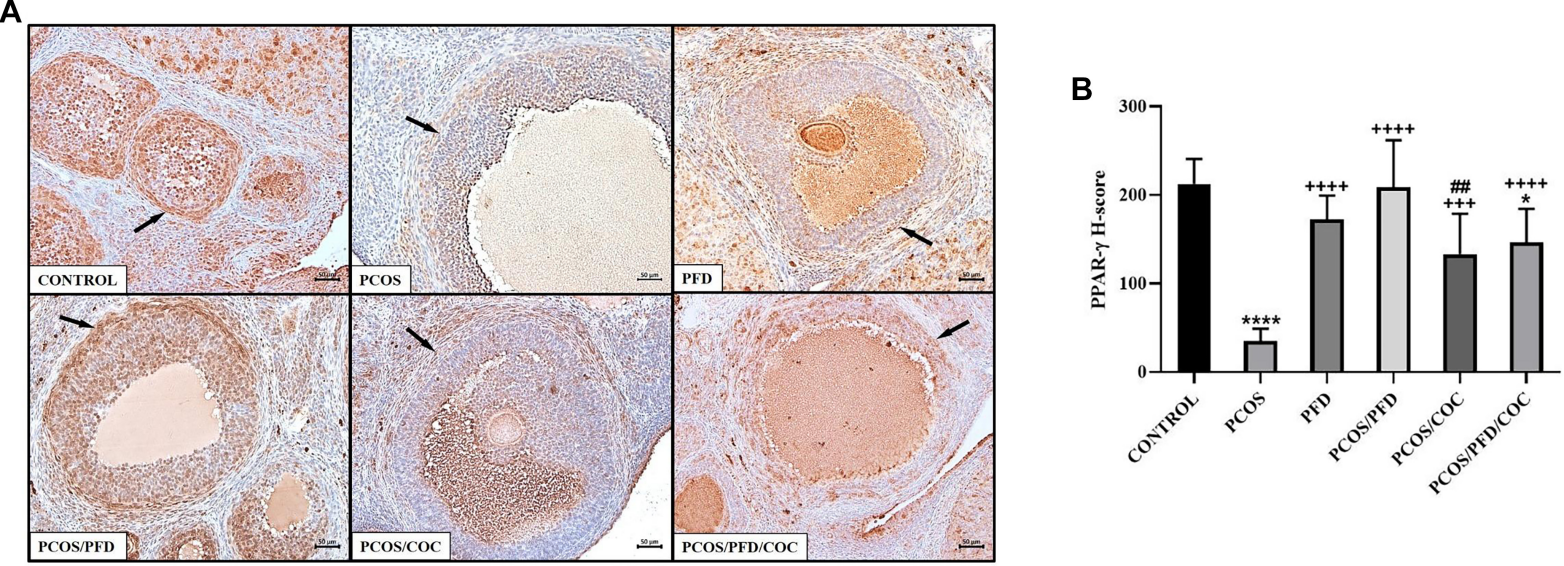
**PFD upregulates PPAR-γ expression in the ovarian stroma of PCOS rats.** (A) Representative immunohistochemical images showing PPAR-γ expression in ovarian sections. Arrows indicate PPAR-γ-positive areas in the stromal tissue surrounding the follicles. Scale bars ═ 200 µm. (B) Comparison of PPAR-γ H-scores between groups (mean ± SD; *n* ═ 7). ^*^P < 0.05, ****P < 0.0001 vs Control; ^+++^*P <* 0.001, ^++++^*P <* 0.0001 vs PCOS; ^##^*P <* 0.01 vs PCOS/PFD. PCOS: Polycystic ovarian syndrome; PFD: Pirfenidone; COC: Combined oral contraceptives; PPAR-γ: Peroxisome proliferator-activated receptor-gamma; SD: Standard deviation.

**Figure 9.**
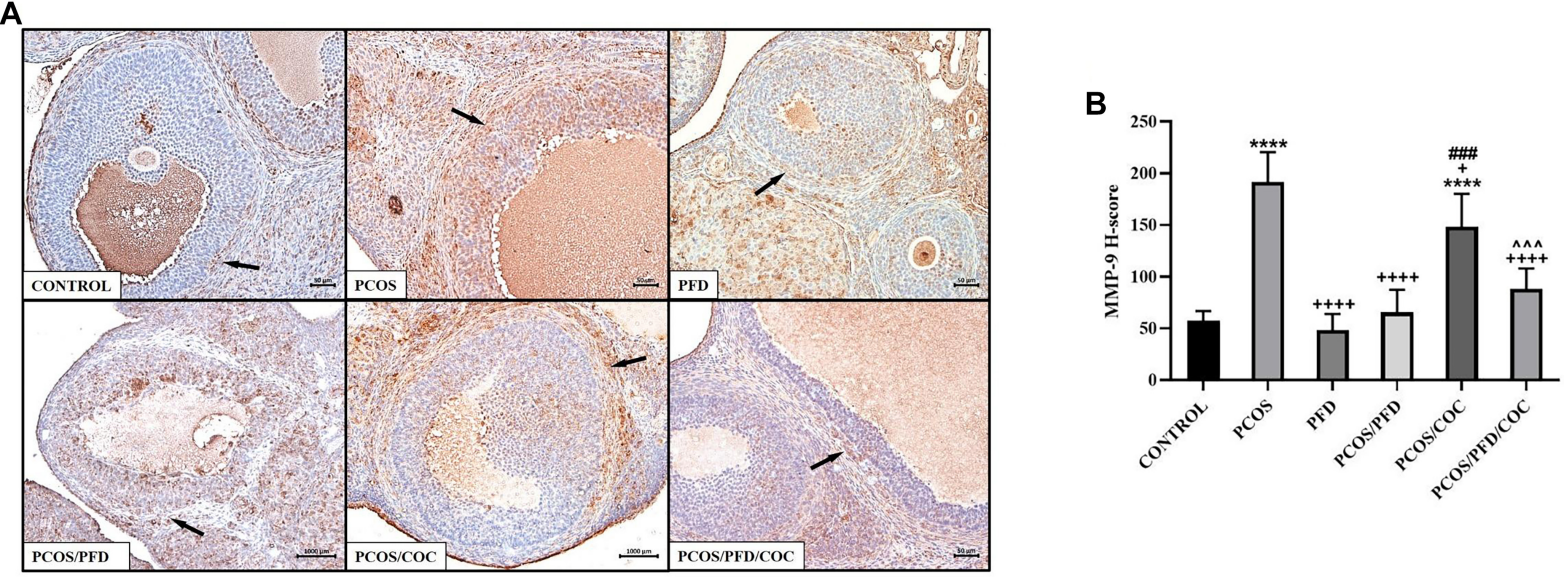
**PFD reduces MMP-9 expression in the ovarian stroma of PCOS rats.** (A) Representative immunohistochemical images showing MMP-9 expression in ovarian sections. Arrows indicate MMP-9-positive areas in the stromal tissue surrounding the follicles. Scale bars ═ 200 µm. (B) Comparison of MMP-9 H-scores between groups (mean ± SD; *n* ═ 7). ****P < 0.0001 vs Control; ^+^*P <* 0.01, ^++++^*P <* 0.0001 vs PCOS; ^###^*P <* 0.001 vs PCOS/PFD; ^ˆˆˆ^*P <* 0.001 vs PCOS/COC. PCOS: Polycystic ovarian syndrome; PFD: Pirfenidone; COC: Combined oral contraceptives; MMP: Matrix metallopeptidase; SD: Standard deviation.

### PFD reduces ovarian fibrosis in PCOS rats

**Figure 10.**
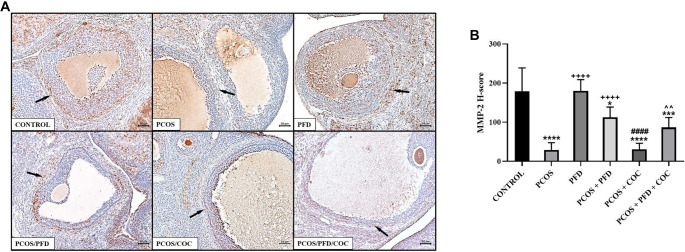
**PFD increases MMP-2 expression in the ovarian stroma of rats with PCOS.** (A) Representative immunohistochemical images showing MMP-2 expression in ovarian sections. Arrows indicate MMP-2-positive areas in the stromal tissue surrounding the follicles. Scale bars ═ 200 µm. (B) Comparison of MMP-2 H-scores between groups (mean ± SD; *n* ═ 7). ^*^P < 0.05, ***P < 0.001, ****P < 0.0001 vs Control; ^++++^*P <* 0.0001 vs PCOS; ^####^*P <* 0.0001 vs PCOS/PFD; ˆˆ*P <* 0.01 vs PCOS/COC. PCOS: Polycystic ovarian syndrome; PFD: Pirfenidone; COC: Combined oral contraceptives; MMP: Matrix metallopeptidase; SD: Standard deviation.

To investigate whether PFD could alleviate ovarian fibrosis in PCOS rats, ovarian tissues were first examined using Masson’s trichrome staining. As shown in Figure 5, the fibrotic area (%) surrounding follicles was significantly increased in the PCOS group (28.89 ± 3.03) compared to the control group (12.45 ± 2.15) (*P* < 0.0001). Treatment with PFD markedly reduced the extent of ovarian fibrosis in PCOS rats (17.04 ± 0.88) (*P* < 0.0001 vs PCOS), suggesting a potential antifibrotic effect. Similarly, COC treatment significantly reduced the fibrotic area relative to the PCOS group (21.56 ± 3.02) (*P* < 0.0001), although the effect was less pronounced than that of PFD (*P* ═ 0.001, PCOS/COC vs PCOS/PFD). Co-administration of PFD and COC produced the most notable improvement (11.76 ± 1.28), closely resembling the control group (*P* ═ 0.9926 vs Control) and demonstrating superior efficacy compared to either monotherapy (*P* ═ 0.002 vs PCOS/PFD; *P* < 0.0001 vs PCOS/COC), indicating a possible additive or synergistic effect. In contrast, PFD administration to healthy rats did not significantly affect fibrotic area compared to untreated controls (11.53 ± 1.15 vs 12.45 ± 2.15; *P* ═ 0.972), reinforcing the specificity of its action under pathological conditions. To assess changes in fibrosis-related protein expression following treatment, immunohistochemical analyses were conducted. As shown in Figure 6, expression of TGF-β1—a key profibrotic factor—was significantly elevated in the stromal compartment of ovaries from PCOS rats (195.83 ± 33.23) compared to controls (12.50 ± 1.73) (*P* < 0.0001). PFD administration in healthy animals did not alter TGF-β1 levels (10.83 ± 11.14; *P* ═ 0.999), while PFD treatment in PCOS rats substantially reduced TGF-β1 expression (48.33 ± 30.11; *P* < 0.0001). Similarly, COC therapy suppressed TGF-β1 expression compared to PCOS (130.00 ± 23.66; *P* < 0.0001), though less effectively than PFD (*P* ═ 0.004 vs PCOS/COC). The combination of PFD and COC further decreased TGF-β1 levels (97.50 ± 30.94), significantly outperforming COC alone (*P* ═ 0.028), but not differing significantly from PFD monotherapy (*P* ═ 0.179). Representative immunohistochemical images and quantitative H-score data are shown in Figure 6A and 6B, respectively. CTGF immunoreactivity was markedly elevated in the PCOS group (177.50 ± 32.37) compared to controls (22.50 ± 9.35; *P* < 0.0001). In healthy animals, PFD administration (28.33 ± 9.83) did not significantly differ from controls (*P* ═ 0.991). In PCOS rats, PFD significantly reduced CTGF expression (73.33 ± 21.37; *P <* 0.0001 vs PCOS). COC treatment (95.00 ± 33.02) and the combination therapy (66.17 ± 12.42) also significantly decreased CTGF levels (*P* < 0.0001 vs PCOS), with combination therapy yielding numerically greater reductions than either monotherapy, though differences were not statistically significant (*P* ═ 0.940 vs PFD; *P* ═ 0.146 vs COC). Representative CTGF staining images are shown in Figure 7A, with H-score comparisons in Figure 7B. PPAR-γ immunoreactivity was significantly decreased in the PCOS group (35.83 ± 14.95) relative to controls (212.50 ± 28.24; *P* < 0.0001). PFD treatment in healthy rats did not significantly affect PPAR-γ levels (172.50 ± 26.79; *P* ═ 0.983). However, in PCOS rats, PFD treatment substantially restored PPAR-γ expression (209.17 ± 52.67; *P* < 0.0001). COC treatment also increased PPAR-γ levels compared to PCOS (133.33 ± 45.57; *P* ═ 0.0001), though less effectively than PFD (*P* ═ 0.001 vs PCOS/PFD). Combined PFD and COC treatment yielded PPAR-γ levels of 146.67 ± 37.77, significantly higher than in the PCOS group (*P* < 0.0001), but not significantly different from PFD monotherapy (*P* ═ 0.197). Figure 8A and 8B displays representative PPAR-γ staining and H-score comparisons, respectively. MMP-9 immunoreactivity was significantly elevated in PCOS rats (191.67 ± 28.75) compared to controls (57.50 ± 9.35; *P* < 0.0001). In healthy animals, PFD treatment (48.33 ± 15.71) showed no significant difference from controls (*P* ═ 0.999). In PCOS rats, PFD significantly reduced MMP-9 expression (65.83 ± 21.54; *P* < 0.0001 vs PCOS). COC treatment also decreased MMP-9 levels (148.33 ± 31.73), though less effectively than PFD (*P* ═ 0.030 vs PCOS; *P* ═ 0.0005 vs PFD). Combination therapy resulted in MMP-9 levels of 88.33 ± 19.66, significantly lower than PCOS (*P* < 0.0001), not significantly different from PFD monotherapy (*P* ═ 0.125), but significantly reduced compared to COC alone (*P* ═ 0.001). Representative MMP-9 staining and group comparisons are presented in Figure 9A and 9B. MMP-2 immunoreactivity was markedly reduced in the PCOS group (29.17 ± 18.28) compared to controls (179.17 ± 59.70) (*P* < 0.0001). In healthy rats, PFD administration (180.00 ± 28.98) did not significantly differ from control levels (*P* ═ 0.997), indicating no effect under normal conditions. In PCOS rats, PFD monotherapy significantly restored MMP-2 expression (112.50 ± 26.22; *P* < 0.0001 vs PCOS). COC treatment (30.83 ± 15.30) failed to affect MMP-2 levels compared to PCOS (*P* ═ 0.868) and was significantly less effective than PFD (*P* < 0.0001). In contrast, the PFD/COC combination therapy significantly upregulated MMP-2 (86.67 ± 25.23), performing better than COC monotherapy (*P* ═ 0.002), though not significantly different from PFD alone (*P* ═ 0.259). Representative images and H-score comparisons are presented in Figure 10A and 10B.

## Discussion

PCOS is increasingly recognized not only as an endocrine and metabolic disorder but also as a fibrotic condition, where ovarian stromal fibrosis plays a significant role in impaired folliculogenesis and ovulatory dysfunction. Excessive accumulation of ECM and collagen—primarily mediated by transforming growth factor-beta (TGF-β) signaling—disrupts normal ovarian architecture and hinders the growth and maturation of follicles [23, 24]. Furthermore, increased deposition of collagen and ECM enhances ovarian stiffness, imposing biomechanical constraints on dominant follicle expansion and oocyte maturation. These mechanisms are supported by both histological and mechanical studies [25]. Animal models and human ovarian tissue studies have demonstrated elevated expression of fibrogenic mediators such as TGF-β1 and CTGF, along with reduced activity of matrix metalloproteinases (MMPs), which together lead to ECM stiffening and pathological stromal remodeling [26–28]. These structural changes create a microenvironment conducive to persistent inflammation and fibrosis, contributing to the chronic course of PCOS. Consequently, targeting ovarian fibrosis has emerged as a promising therapeutic strategy to restore normal follicular development and improve ovulatory outcomes in affected individuals. Histological analysis in our study confirmed that letrozole-induced PCOS resulted in typical polycystic morphology, including an increased number of cystic follicles, reduced granulosa layers, and a marked decrease in corpora lutea. PFD administration in PCOS rats significantly improved all key morphological parameters. COC therapy similarly led to partial morphological improvements; however, the number of corpora lutea remained relatively low—consistent with its known pharmacological action of suppressing ovulation rather than indicating inadequate follicular development. This distinction is important, as COC’s mechanism may limit the appearance of corpora lutea despite improvements in follicle structure. Notably, the combination of PFD and COC produced the most balanced histological profile, suggesting a potential additive effect, particularly in restoring follicular heterogeneity and reducing cystic transformation. Despite the successful induction of PCOS-related ovarian changes, no significant alterations in systemic estrogen or testosterone levels were observed across experimental groups. This aligns with previous studies reporting that letrozole-induced PCOS models may not always produce marked elevations in circulating androgens, even in the presence of clear morphological and functional disruptions [29]. The absence of a hormonal response to treatment may reflect that a pathological increase in androgen levels was not robustly established in the disease model. Notably, both PFD and COC treatments improved estrous cyclicity and follicular morphology without affecting systemic hormone levels, suggesting that restoration of ovulatory function may not necessarily require normalization of endocrine parameters. Our findings are consistent with a recent study showing that chronic administration of PFD over 8.5 and 12 months significantly increased follicle and corpus luteum numbers in aged mice, while estradiol levels remained unchanged [16]. Thus, we propose that targeting intraovarian fibrosis may offer a viable alternative or complement to traditional hormonal therapies in managing PCOS-related ovulatory dysfunction. Our histological and immunohistochemical analyses revealed that PFD treatment significantly attenuated ovarian fibrosis in the PCOS model, as demonstrated by reduced collagen deposition on Masson’s trichrome staining and downregulation of key fibrotic markers, including TGF-β1, CTGF, and MMP-9. These results align with prior reports indicating that overexpression of TGF-β1 and CTGF contributes to aberrant ECM accumulation and stromal remodeling in polycystic ovaries, ultimately impairing folliculogenesis [29, 30]. Importantly, PFD restored PPAR-γ expression—a transcription factor known to inhibit fibrogenic pathways and antagonize TGF-β activity—consistent with its established anti-fibrotic role in reproductive tissues [31, 32]. Moreover, the upregulation of MMP-2 following PFD treatment suggests reactivation of ECM degradation mechanisms, supporting tissue remodeling [33]. While the combined PFD and COC treatment yielded the most comprehensive histological improvements, monotherapy with PFD alone was sufficient to normalize stromal architecture and reduce fibrosis to near-control levels. These findings support the idea that antifibrotic therapy, independent of hormonal modulation, can restore ovarian structure in PCOS—underscoring the pathogenic significance of fibrosis and its potential as a therapeutic target. Importantly, the PFD dose used in our study is translatable to clinical settings. Using a standard BSA-based interspecies conversion factor (∼6.2), the rat NOAEL of 200 mg/kg corresponds to approximately 32 mg/kg in humans, or ∼2240 mg/day for a 70 kg adult, which is close to the approved clinical dose of 2403 mg/day [33, 34]. Therefore, the dosage was considered both experimentally validated and pharmacologically relevant for evaluating antifibrotic outcomes in ovarian tissue of PCOS rats. Although this study focused primarily on the ovarian fibrotic aspect of PCOS, it is important to recognize the broader metabolic disturbances often associated with the syndrome, such as insulin resistance and dyslipidemia. While we did not directly assess metabolic parameters in this study, prior research suggests that PFD may exert beneficial effects on systemic metabolic functions. For instance, in rodent models of metabolic-associated fatty liver disease (MAFLD) and obesity-related insulin resistance, PFD administration improved insulin sensitivity, reduced hepatic steatosis, and attenuated pro-inflammatory signaling pathways [35, 36]. These findings indicate that PFD’s antifibrotic actions may extend beyond local tissue effects to broader metabolic benefits. Given the strong link between ovarian fibrosis and systemic metabolic dysfunction in PCOS, future studies should incorporate evaluations of glucose homeostasis, lipid metabolism, and insulin signaling to more comprehensively assess the therapeutic potential of PFD in addressing both reproductive and metabolic features of the syndrome.

Despite the promising findings, several limitations of our study should be acknowledged. First, the relatively small sample size (*n* ═ 7 per group) may have limited our ability to detect subtle biological differences, particularly in hormone levels and outcomes within the combination therapy group. Larger cohorts will be necessary in future studies to validate and strengthen these observations. Second, the 21-day treatment duration may not fully capture the durability of antifibrotic effects or the potential long-term systemic consequences. Longitudinal studies are therefore needed to assess sustained efficacy and safety. Although we observed significant changes in key fibrosis mediators such as TGF-β1, CTGF, PPAR-γ, and MMPs, we did not investigate downstream signaling pathways (e.g., Smad2/3, PI3K/Akt) or cellular mechanisms using *in vitro* models. Mechanistic studies involving granulosa or stromal cell cultures could clarify how PFD directly modulates fibrotic remodeling in ovarian tissue. Third, the clear morphological and functional improvements observed in the absence of significant changes in systemic sex hormone levels raise an important question: are the therapeutic effects of PFD primarily mediated by local intraovarian mechanisms rather than endocrine modulation? Our findings suggest that stromal remodeling alone may be sufficient to restore follicular dynamics. However, this hypothesis warrants further investigation, including measurements of LH, FSH, intraovarian androgens, and metabolic parameters such as insulin sensitivity and lipid profiles. Lastly, while PFD’s antifibrotic effects are well established, its roles in regulating oxidative stress and inflammation were not addressed in this study. Future research should explore these aspects to better define the multifactorial nature of PFD’s therapeutic actions.

## Conclusion

In conclusion, this is the first study to demonstrate the therapeutic potential of PFD in PCOS-associated ovarian fibrosis, highlighting a novel and previously unexplored avenue for treatment. Our findings indicate that ovarian fibrosis is a key pathological feature in the letrozole-induced experimental model of PCOS, contributing to impaired follicular development and ovulatory dysfunction. Treatment with the antifibrotic agent PFD significantly improved ovarian morphology and function by modulating the TGF-β1 signaling pathway. This led to reduced fibrosis, improved estrous cyclicity, and a decrease in cystic follicle formation—hallmark features of PCOS. Although COCs, a standard treatment option, provided partial improvement in fibrotic remodeling, their effects were less pronounced than those of PFD. Taken together, our findings suggest that PFD’s antifibrotic activity in ovarian tissue offers a promising non-hormonal therapeutic strategy to restore follicular function in PCOS. Targeting stromal remodeling and fibrosis may represent a new direction for future treatments that go beyond conventional endocrine modulation.

## Data Availability

All data supporting the results of the present study are included in the article.
